# Blockage of indoleamine 2,3-dioxygenase regulates Japanese encephalitis via enhancement of type I/II IFN innate and adaptive T-cell responses

**DOI:** 10.1186/s12974-016-0551-5

**Published:** 2016-04-18

**Authors:** Seong Bum Kim, Jin Young Choi, Erdenebileg Uyangaa, Ajit Mahadev Patil, Ferdaus Mohd Altaf Hossain, Jin Hur, Sang-Youel Park, John-Hwa Lee, Koanhoi Kim, Seong Kug Eo

**Affiliations:** College of Veterinary Medicine and Bio-Safety Research Institute, Chonbuk National University, Iksan, 54596 Republic of Korea; Department of Bioactive Material Sciences, Graduate School, Chonbuk National University, Jeonju, 54896 Republic of Korea; Department of Pharmacology, School of Medicine, Pusan National University, Yangsan, 50612 Republic of Korea

**Keywords:** Indoleamine 2,3-dioxygenase, Japanese encephalitis, Neuroinflammation, Type I/II interferons

## Abstract

**Background:**

Japanese encephalitis (JE), a leading cause of viral encephalitis, is characterized by extensive neuroinflammation following infection with neurotropic JE virus (JEV). Indoleamine 2,3-dioxygenase (IDO) has been identified as an enzyme associated with immunoregulatory function. Although the regulatory role of IDO in viral replication has been postulated, the in vivo role of IDO activity has not been fully addressed in neurotropic virus-caused encephalitis.

**Methods:**

Mice in which IDO activity was inhibited by genetic ablation or using a specific inhibitor were examined for mortality and clinical signs after infection. Neuroinflammation was evaluated by central nervous system (CNS) infiltration of leukocytes and cytokine expression. IDO expression, viral burden, JEV-specific T-cell, and type I/II interferon (IFN-I/II) innate responses were also analyzed.

**Results:**

Elevated expression of IDO activity in myeloid and neuron cells of the lymphoid and CNS tissues was closely associated with clinical signs of JE. Furthermore, inhibition of IDO activity enhanced resistance to JE, reduced the viral burden in lymphoid and CNS tissues, and resulted in early and increased CNS infiltration by Ly-6C^hi^ monocytes, NK, CD4^+^, and CD8^+^ T-cells. JE amelioration in IDO-ablated mice was also associated with enhanced NK and JEV-specific T-cell responses. More interestingly, IDO ablation induced rapid enhancement of type I IFN (IFN-I) innate responses in CD11c^+^ dendritic cells (DCs), including conventional and plasmacytoid DCs, following JEV infection. This enhanced IFN-I innate response in IDO-ablated CD11c^+^ DCs was coupled with strong induction of PRRs (RIG-I, MDA5), transcription factors (IRF7, STAT1), and antiviral ISG genes (Mx1, Mx2, ISG49, ISG54, ISG56). IDO ablation also enhanced the IFN-I innate response in neuron cells, which may delay the spread of virus in the CNS. Finally, we identified that IDO ablation in myeloid cells derived from hematopoietic stem cells (HSCs) dominantly contributed to JE amelioration and that HSC-derived leukocytes played a key role in the enhanced IFN-I innate responses in the IDO-ablated environment.

**Conclusions:**

Inhibition of IDO activity ameliorated JE via enhancement of antiviral IFN-I/II innate and adaptive T-cell responses and increased CNS infiltration of peripheral leukocytes. Therefore, our data provide valuable insight into the use of IDO inhibition by specific inhibitors as a promising tool for therapeutic and prophylactic strategies against viral encephalitis caused by neurotropic viruses.

## Background

Japanese encephalitis (JE) is an acute zoonotic, mosquito-borne disease caused by JE virus (JEV), a single-stranded, positive-sense RNA (~11 kb, monopartite, linear) virus belonging to the family Flaviviridae and the genus *Flavivirus* [1]. Infection with neurotropic flaviviruses of the JE serotype, which include JE, Murray Valley encephalitis, St. Louis encephalitis, and West Nile virus (WNV), results in debilitating neurological disorders in a significant proportion of clinical cases [2, 3]. JE is a leading cause of viral encephalitis manifested by extensive neuroinflammation in the central nervous system (CNS) and disruption of the blood-brain barrier (BBB). In humans, the clinical presentation of JEV infection ranges from mild febrile illness to severe meningoencephalitis [4]. Due to rapid changes in climate and demography, vector-transmitted JE poses an increasing threat to global health and welfare with nearly 70,000 cases reported annually [5–7]. The incubation period of JE ranges from 5 to 15 days, and most JEV infections in endemic regions manifest as mild febrile, subclinical disease which leads to protective adaptive immune responses [4]. However, approximately 25–30 % of JE cases, mostly in infants, are lethal and 50 % of cases result in permanent neuropsychiatric sequelae [4]. Thus, JE is considered more fatal than encephalitis caused by WNV infection, which has a fatality rate of 3–5 % (1100 deaths/29,000 symptomatic infections) [7, 8]. Currently, more than 60 % of the world’s population inhabits JE endemic areas, such as eastern and southern Asia, and the virus is spreading to previously unaffected regions, including Indonesia, Pakistan, and northern Australia [5, 6]. However, despite the importance of JE, little is known regarding potential therapeutic strategies for regulating JE progression.

Indoleamine 2,3-dioxygenase (IDO) has been identified as an enzyme associated with powerful immunoregulatory function, likely derived from its enzymatic activity, which leads to catabolism of the essential amino acid l-tryptophan (l-TRP) [9–14]. Therefore, IDO-mediated depletion of _L_-TRP and the resulting metabolites (l-kynurenine, l-KYN) induces an immunosuppressive environment through provoking tolerogenicity of antigen-presenting cells (APCs), T-cell anergy, and immune cell death [9, 10]. IDO can be induced in a variety of cell types, including dendritic cells (DCs) [15], macrophages [16], and epithelial cells [17]. These cell types play an important role in controlling viral replication and facilitating antigen-specific adaptive immune responses [9, 10]. In various tissues, IDO activity has been induced by several cytokines after viral infection, and its enzymatic activity can be blocked using the pharmacological competitive inhibitor, 1-methyl-d,l-tryptophan (1-MT) [18]. Thus, inhibition of IDO with the competitive inhibitor 1-MT may be a promising strategy for enhancing immune responses in various viral infection models, including human immunodeficiency virus (HIV) and influenza virus [19, 20]. Also, IDO ablation was shown to suppress viral replication through the upregulation of type I interferon (IFN-I) production in a retrovirus-infected murine model [21]. Although the regulatory role of IDO in viral replication has been reported in a few studies using an in vivo viral infection model, the in vivo role of IDO activity is not fully understood in viral encephalitis caused by infection with neurotropic viruses such as JEV and WNV.

The molecular pathogenesis of viral encephalitis, including JE, remains unclear. However, JE is considered an immunopathological disease because CNS invasion by JEV drives the stimulation of microglia/glia and infiltrating leukocytes, leading to indirect death of neuron cells via the uncontrolled secretion of pro-inflammatory cytokines (such as IL-6 and TNF-α) and soluble mediators [22, 23]. Furthermore, JEV infects and kills neuron cells directly in the CNS. This complicated progression of JE after viral infection in the host prompted us to explore the role of IDO in JE progression. Here, we found that IDO expression in myeloid cells and in neurons of the lymphoid and CNS tissues was closely associated with clinical signs of JE. Furthermore, the inhibition of IDO activity using genetic ablation or 1-MT provided enhanced resistance to JE, along with a reduced viral burden and the early and increased CNS infiltration of myeloid and lymphoid leukocytes. JE amelioration was also associated with enhanced NK and antigen-specific T-cell responses. More interestingly, the rapid enhancement of IFN-I innate immune responses in CD11c^+^ DCs (conventional and plasmacytoid DCs) and neuron cells likely contributed to the protective role of IDO ablation in JE. Therefore, our data suggest that the inhibition of IDO with pharmacological inhibitors could be a promising therapeutic strategy for regulating JE progression.

## Methods

### Ethics statement

All animal experiments described in the present study were conducted at Chonbuk National University according to the guidelines set by the Institutional Animal Care and Use Committees (IACUC) of Chonbuk National University and were pre-approved by the Ethical Committee for Animal Experiments of Chonbuk National University (permission code 2013-0028). The animal research protocol used in this study followed the guidelines set up by the nationally recognized Korea Association for Laboratory Animal Sciences (KALAS). All experimental protocols requiring biosafety were approved by Institutional Biosafety Committees (IBC) of Chonbuk National University.

### Animals, cells, and viruses

C57BL/6 (H-2^b^) mice (4–5 weeks old) were purchased from Samtako (O-San, Korea). IDO (H-2^b^) knockout (KO) mice were obtained from The Jackson Laboratory (Bar Harbor, ME, USA). All mice were genotyped and bred in the animal facilities of Chonbuk National University. JEV Beijing-1 strain was obtained from Green Cross Research Institute (Suwon, Korea) and propagated in the mosquito cell line, C6/36, using DMEM supplemented with 2 % FBS, penicillin (100 U/ml), and streptomycin (100 U/ml) [24]. The C6/36 cultures were infected with JEV Beijing-1 at a multiplicity of infection (MOI) of 0.1 and were incubated in a humidified CO_2_ incubator for 1 h at 28 °C. After absorption, the inoculum was removed, and 7 ml of a maintenance medium containing 2 % FBS was added. Approximately 6–7 days post-infection (dpi), cultures of host cells showing an 80–90 % cytopathic effect were harvested. The virus stocks were titrated using either a conventional plaque assay or a focus-forming assay and were stored in aliquots at −80 °C until use.

### Antibodies and reagents

The mAbs used for flow cytometric analyses and other experiments were obtained from eBioscience (San Diego, CA, USA) or R&D Systems (Minneapolis, MN, USA): fluorescein isothiocyanate (FITC)-conjugated anti-CD4 (RM4-5), CD8 (53-6.7), Ly-6G (1A8), B220 (RA3-6B2); phycoerythrin (PE)-conjugated anti-mouse-CD11b (M1/70), Foxp3 (FJK-16s), CD154 (MR1); peridinin chlorophyll protein complex (PerCP)-conjugated anti-Ly-6C (HK1.4); PE-cyanine dye (Cy5.5)-conjugated anti-mouse IFN-γ (XMG1.2); PE-cyanine dye (Cy7)-conjugated anti-mouse NK1.1 (PK136), IL-5 (TRFK4); and allophycocyanin (APC)-conjugated anti-mouse-CD3ε (145-2c11), CD25 (PC62.5), CD49b (DX5), Ly-6G (Gr-1), TNF-α (MP6-XT22), IL-4 (BVD6-24G2), and IL-17A (eBio17B7). The peptides of I-A^b^-restricted epitopes (JEV NS1_132–145_ [TFVVDGPETKECPD] and NS3_563–574_ [WCFDGPRTNAIL]) and H-2D^b^-restricted epitopes (JEV NS4B_215–223_ [SAVWNSTTA]) were chemically synthesized at Peptron Inc. (Daejeon, Korea). JEV-specific primers for the detection of viral RNA (JEV10,564–10,583 forward, 5′-CCC TCA GAA CCG TCT CGG AA-3′ and JEV10,862–10,886 reverse, 5′-CTA TTC CCA GGT GTC AAT ATG CTG T-3′) and primers specific for cytokines, IFN-I (IFN-α/β), and RLRs, IRFs, ISGs (Table 1) were synthesized at Bioneer Corp. (Daejeon, Korea) and used for PCR amplification of target genes.

**Table 1:** Specific primers for cytokines, chemokines, type I IFNs, and ISGs used in real-time qRT-PCR

Gene name	Primer sequence (5′-3′)	Position cDNA	GeneBank ID
IL-6	FP: TGG GAA ATC GTG GAA ATG AG RP: CTC TGA AGG ACT CTG GCT TTG	209–228 442–462	NM_031168
TNF-α	FP: CGT CGT AGC AAA CCA CCA AG RP: TTG AAG AGA ACC TGG GAG TAG ACA	438–457 564–587	NM_013693
IFN-α	FP: TGTCTGATGCAGCAGGTGG RP: AAGACAGGGCTCTCCAGAC	367–385 514–532	NM_008334.3
IFN-β	FP: TCCAAGAAAGGACGAACATTCG RP: TGAGGACATCTCCCACGTCAA	106–121 399–419	NM_010510
CCL2	FP: AAA AAC CTG GAT CGG AAC CAA RP: CGG GTC AAC TTC ACA TTC AAA G	347–367 426–447	NM_011333
CCL3	FP: CCA AGT CTT CTC AGC GCC AT RP: GAA TCT TCC GGC TGT AGG AGA AG	158–177 206–228	NM_011337.2
CCL4	FP: TTC TGT GCT CCA GGG TTC TC RP: GAG GAG GCC TCT CCT GAA GT	128–147 388–407	NM_013652.2
CCL5	FP: CCC TCA CCA TCA TCC TCA CT RP: CTT CTT CTC TGG GTT GGC AC	77–96 275–294	NM_013653.3
IRF3	FP: GAT GGA GAG GTC CAC AAG GA RP: GAG TGT AGC GTG GGG AGT GT	1170–1189 1259–1278	NM_016849
IRF5	FP: GGA AGA AAT GAA GCC AGC AG RP: ACC CTG GGG TAA TTG GAC TC	1931–1950 2001–2020	NM_001252382.1
IRF7	FP: CCT CTT GCT TCA GGT TCT GC RP: GCT GCA TAG GGT TCC TCG TA	980–999 1080–1099	NM_016850.3
RIG-I	FP: CCA CCT ACA TCC TCA GCT ACA TGA RP: TGG GCC CTT GTT GTT CTT CT	194–217 260–279	NM_172689
MDA5	FP: GGC ACC ATG GGA AGT GAT T RP: ATT TGG TAA GGC CTG AGC TG	1178–1196 1247–1266	NM_027835
PKR	FP: AAC TTC TTC ACA CGT GCT TC RP: CAT TCA GCC AAG GTC TTC AG	1517–1536 1678–1697	NM_011163
STAT1	FP: AAG CGA ACT GGA TAC ATC A RP: CCG GGA CAT CTC ATC AAA C	2093–2111 2194–2212	U06924.1
2′5′-Oas1	FP: CCA TCC TCA AGT GGA CAA GAA CTG RP: TTG GGC TTT GGG CAC CTT C	1235–1258 1359–1377	AF466822.1
Oasl-1	FP: CCA GGA AGA AGC CAA GCA CCA TC RP: AGG TTA CTG AGC CCA AGG TCC ATC	377–399 449–472	NM_145209.3
Mx1	FP: CAG CAC CTG ATG GCC TAT CA RP: ACG TCT GGA GCA TGA AGA ACT G	2276–2295 2363–2342	NM_010846.1
Mx2	FP: AGG CTC ACA ACC GCA TCT RP: GCT CAG CAA ACA TTT TCA GG	1858–1875 1910–1929	NM_013606.1
ISG49	FP: GCC GTT ACA GGG AAA TAC TGG RP: CCT CAA CAT CGG GGC TCT	919–939 1126–1143	NM_010501.2
ISG54	FP: GGG AAA GCA GAG GAA ATC AA RP: TGA AAG TTG CCA TAC AGA AG	1918–1937 2005–2024	NM_008332.3
ISG56	FP: CAG AAG CAC ACA TTG AAG AA RP: TGT AAG TAG CCA GAG GAA GG	774–793 911–930	NM_008331.3
β-actin	FP: TGG AAT CCC TGT GGG ACC ATG AAA C RP: TAA AAC GCA GCT CAG TAA CAG TCC G	885–909 1209–1233	NM_007393.3

*IL* interleukin, *TNF-*α tumor necrosis factor-α, *IFN* interferon, *FP* forward primer, *RP* reverse primer

### Quantitative real-time RT-PCR for viral burden and cytokine expression

Viral burden and the expression of cytokines (IL-6, TNF-α, and IFN-α/β), CC chemokines, and IDO in inflammatory and lymphoid tissues were determined by conducting quantitative SYBR Green-based real-time RT-PCR (real-time qRT-PCR). Mice were infected intraperitoneally (i.p.) with JEV (3.0 × 10^7^ plaque-forming unit (PFU)) and tissues, including brain, spinal cord, and spleen, were harvested at 2 and 4 dpi following extensive cardiac perfusion with Hank’s balanced salt solution (HBSS). Total RNA was extracted from tissues using easy-BLUE (iNtRON, Inc., Daejeon, Korea) and subjected to real-time qRT-PCR using a CFX96 Real-Time PCR Detection system (Bio-Rad Laboratories, Hercules, CA, USA). Following reverse transcription of total RNA with the High-Capacity cDNA Reverse Transcription Kits (Applied Biosystems, Foster, CA, USA), the reaction mixture contained 2 μl of template cDNA, 10 μl of 2× SYBR Premix Ex Taq, and 200 nM primers, yielding a final volume of 20 μl. The reactions were denatured at 95 °C for 30 s and then subjected to 45 cycles of 95 °C for 5 s and 60 °C for 20 s. After the reaction cycle was complete, the temperature was increased from 65 to 95 °C at a rate of 0.2 °C/15 s, and the fluorescence was measured every 5 s to construct a melting curve. A control sample that contained no template DNA was run with each assay, and all determinations were performed at least in duplicates to ensure reproducibility. The authenticity of the amplified product was determined by melting curve analysis. All data were analyzed using the Bio-Rad CFX Manager, version 2.1 analysis software (Bio-Rad Laboratories). Viral burden was expressed by the copy number of viral RNA per microgram of total RNA, after calculating the absolute copy number of viral RNA in comparison with the standard cDNA template of viral RNA.

### Determination of l-kynurenine levels in the sera and brain

The levels of l-kynurenine in the sera and brain homogenates following JEV infection were measured via HPLC after deproteination using a C18 reverse phase column [25]. The levels of l-kynurenine in the sera and brain homogenates were expressed as micromolars and picomoles per milligram tissue, respectively. Levels of l-kynurenine were used for an in vivo index of IDO enzyme activity.

### Analysis of leukocyte infiltration into the CNS

Mice infected with JEV were perfused with 30 ml of HBSS at 3 or 5 dpi via cardiac puncture of the left ventricle. Brains were then harvested and homogenized by gently pressing them through a 100-mesh tissue sieve, after which they were digested with 25 μg/ml collagenase type IV (Worthington Biochem, Freehold, NJ, USA), 0.1 μg/ml trypsin inhibitor *N*α-*p*-tosyl-l-lysine chloromethyl ketone, 10 μg/ml DNase I (Amresco, Solon, OH, USA), and 10 mM HEPE in HBSS for 1 h at 37 °C, under shaking conditions. Cells were separated using an Optiprep density gradient (18/10/5 %) with centrifugation at 800×*g* for 30 min (Axis-Shield, Oslo, Norway), after which cells were collected from the 18 to the 10 % interface and washed twice with PBS. Cells were counted and stained for CD11b, Ly6G, Ly6C, CD3ε, CD4, CD8α, DX5, and NK1.1 with directly conjugated antibodies (eBioscience) for 30 min at 4 °C. Finally, the cells were fixed with 10 % formaldehyde. Data collection and analysis were performed with a FACSCalibur flow cytometer (Becton Dickson Medical Systems Sharon, MA, USA) and FlowJo (Tree Star, San Carlos, CA, USA) software.

### Analysis and activation of NK cells

The activity of NK cells was assessed by their capacity to produce IFN-γ and granzyme B (GrB) following brief stimulation with PMA and ionomycin (Sigma-Aldrich, St. Louis, MO, USA). Splenocytes were prepared from BL/6 and IDO KO mice 2 dpi and stimulated with PMA (50 ng/ml) and ionomycin (750 ng/ml) for 1 and 2 h, respectively, in the presence of monensin (2 μM) to induce the accumulation of IFN-γ and GrB. After stimulation, cells were surface-stained with FITC anti-mouse-CD3ε, PE-Cy7 anti-mouse NK1.1, biotin-conjugated anti-mouse pan-NK cells (CD49b) [DX5] antibodies, and streptavidin-APC for 30 min at 4 °C. Cells were then washed twice with FACS buffer containing monensin. After fixation, the cells were permeabilized with 1× permeabilization buffer (eBioscience) and stained intracellularly with PE anti-mouse IFN-γ (XMF1.2) and GrB antibodies in permeabilization buffer for 30 min at 4 °C. Finally, the cells were washed twice with PBS, and analysis was performed with a FACSCalibur flow cytometer (Becton Dickson Medical Systems) and FlowJo software (ver. 7.6.5; Tree Star).

### JEV-specific CD4^+^ and CD8^+^ T-cell responses

JEV-specific CD4^+^ and CD8^+^ T-cell responses were determined by intracellular CD154 [26, 27], IFN-γ, and TNF-α staining in response to stimulation with JEV epitope peptides. Surviving mice infected with 3.0 × 10^7^ PFU JEV were sacrificed at 7 dpi, and splenocytes were prepared. The erythrocytes were depleted by treating single-cell suspensions with ammonium chloride-containing Tris buffer (NH_4_Cl-Tris) for 5 min at 37 °C. The splenocytes were cultured in 96-well culture plates (5 × 10^5^ cells/well) in the presence of synthetic peptide epitopes (NS1_132–145_, NS3_563–574_, and NS4B_215–225_) for 12 and 6 h, in order to observe CD4^+^ and CD8^+^ T-cell responses, respectively. Monensin at a concentration of 2 μM was added to antigen-stimulated cells 6 h before harvest. Cells were washed twice with PBS and surface-stained with FITC-anti-CD4 or CD8 antibodies for 30 min at 4 °C, and then washed twice with PBS containing monensin. After fixation, the cells were washed twice with permeabilization buffer (eBioscience) and stained with PE Cy5.5-anti-IFN-γ or APC-anti-TNF-α in permeabilization buffer for 30 min at room temperature. Intracellular CD154 was detected by the addition of CD154 mAb to culture media during peptide stimulation. Finally, the cells were washed twice with PBS and fixed using fixation buffer. Sample analysis was performed with a FACSCalibur flow cytometer (Becton Dickson Medical Systems) and FlowJo (Tree Star) software.

### Intracellular staining for analysis of CD4^+^ Th1, Th17, and Treg cells

To monitor CD4^+^ Th subsets, mice were infected i.p. with 3.0 × 10^7^ PFU of JEV, sacrificed at 5 dpi, and splenocytes were prepared. Splenocytes were then cultured in 96-well culture plates (10^6^ cells/well) with PMA/Ionomycin (Th1 and Th17) in the presence of monensin (2 μM) for 5 h at 37 °C. The stimulated cells were washed twice with PBS and surface stained with FITC-anti-CD4 for 30 min at 4 °C and then washed twice with PBS containing monensin. After fixation, the cells were washed twice with permeabilization buffer (eBioscience) and stained with PerCP-anti-IFN-γ and APC-anti-IL-17α in permeabilization buffer for 30 min at room temperature. Finally, the cells were washed twice with PBS and fixed using fixation buffer. To monitor Treg cells, splenocytes were stained by surface staining for FITC-anti-CD4 markers for 30 min on ice and then fixed with fixation/permeabilization concentrate buffer (eBioscience) for 6 h at 4 °C. After fixation, the cells were washed twice with permeabilization buffer (eBioscience) and stained with PE-anti-Foxp3 in permeabilization buffer for 30 min at room temperature. The sample analysis was performed using a FACSCalibur flow cytometer.

### Primary cell culture and infection

#### Myeloid-derived DCs and macrophages

Bone marrow-derived conventional DCs (BMDCs), plasmacytoid DCs (pDCs), and macrophages (BMDMs) were prepared from BM cells from BL/6 and IDO KO mice, as described earlier with some modifications [24]. Briefly, for BMDCs and pDCs, BM cells (3 × 10^5^ cells/ml) from femurs and tibiae were cultured in RPMI 1640 supplemented with GM-CSF (2 ng/ml) plus IL-4 (10 ng/ml) and Flt3-L (10 ng/ml), respectively. On day 3, another 6 ml of fresh complete medium containing GM-CSF plus IL-4 and Flt3-L was added, and half of the medium was changed on day 6. On day 8, non-adherent and loosely adherent DCs were harvested by vigorous pipetting. Cells were then characterized by flow cytometric analysis, which revealed that the culture generally consisted of >85 % CD11c^+^ cells (25 % CD11c^+^CD11b^+^ and 65 % CD11c^+^CD8α^+^), and >90 % CD11c^+^PDCA-1^+^B220^+^. BMDMs were prepared by culturing bone marrow cells in DMEM supplemented with 30 % L929 cell-conditioned medium (LCCM) as a source of macrophage colony-stimulating factor (M-CSF). On day 3, another 6 ml of fresh complete medium containing 30 % LCCM was added, and half of the medium was changed on day 6. The cultured cells were harvested following an 8-day incubation and were analyzed by flow cytometry. The prepared BMDMs were composed of >85 % F4/80^+^ cells that consisted of 99.2 % F4/80^+^CD11b^+^ and ~1 % F4/80^+^CD11c^+^ cells. Prepared BMDCs, pDCs, and BMDMs were infected with JEV at MOIs of 0.1, 1.0, and 10 for analysis of viral replication and 10 MOI for evaluating cytokine expression.

#### Primary microglia

Primary microglia cells were recovered from the brains of fetal BL/6 and IDO KO mice (1–3 days old), as described previously [28]. Primary microglia cells recovered from the brains via trypsin + EDTA digestion were initially seeded on poly-d-lysine/laminin-coated plates in DMEM containing 10 % FBS and mechanically isolated from glia cells by a brief duration of shaking (100 rpm, 1 h). Primary microglia cells were infected with JEV at MOIs of 1.0 and 10 for analysis of viral replication and 10 MOI for the evaluation of cytokine, IFN-I, RLR, and ISG gene expression.

#### Primary cortical neurons

Primary cortical neurons were prepared from 15-day-old embryos, as described previously [24]. Cortical neurons were seeded in 12-well poly-d-lysine/laminin-coated plates in DMEM containing 5 % FBS and 5 % horse serum for 24 h, and then cultured for 4 days with Neurobasal medium containing B27 supplement and l-glutamine (Invitrogen, Carlsbad, CA, USA). Primary cortical neurons were infected with JEV at MOIs of 0.001 and 0.01 for analysis of viral replication and 0.1 MOI for the evaluation of cytokine expression.

### Generation of BM chimeric mice and determination of serum IFN-β

BL/6 mice (5 weeks old) and IDO KO mice were irradiated with one dose of 900 rads. Within 12 h, recipient mice were reconstituted with 10^7^ donor BM cells derived from BL/6 and IDO KO mice. The recipient mice were given sulfamethoxazole and trimethoprim in their drinking water for 10 days after irradiation. Mice were infected with JEV 4–6 weeks after irradiation. A commercial ELISA kit (PBL Biomedical Laboratories, Piscataway, NJ, USA) was used to measure levels of secreted IFN-β protein in sera according to the manufacturer’s protocol.

### Statistical analysis

All data were expressed as the average ± standard deviation, and statistically significant differences between groups were analyzed by unpaired two-tailed Student’s *t* tests for ex vivo experiments and immune cell analyses or ANOVA and post hoc test for multiple comparisons of the mean. The statistical significance of viral burden was evaluated using the Mann-Whitney test or unpaired two-tailed Student’s *t* test. Kaplan-Meier survival curves were analyzed with the log-rank test. A *p* value of ≤0.05 was considered significant. All data were analyzed using Prism software (GraphPad Prism4, San Diego, CA, USA).

## Results

### IDO expression correlates with clinical signs of JE

**Fig. 1:**
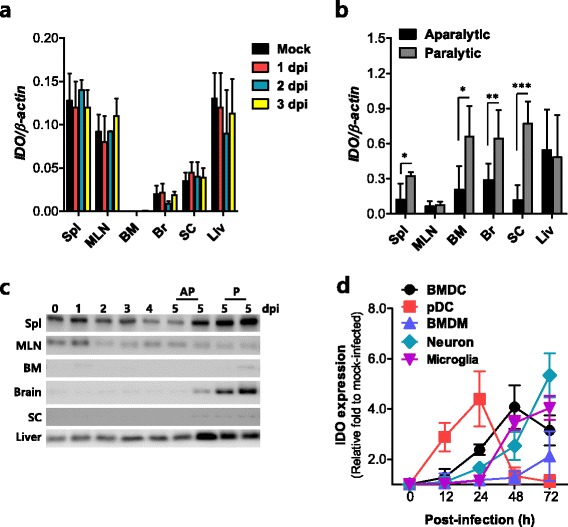
IDO expression is correlated with clinical signs of JE. **a** IDO expression at the early stage of JE. IDO expression was determined by real-time qRT-PCR using total RNA extracted from several tissues, including the spleen (*Spl*), mesenteric LN (*MLN*), bone marrow (*BM*), brain (*Br*), spinal cord (*SC*), and liver (*Liv*), 1, 2, and 3 days following JEV infection. **b** Comparison of IDO expression between aparalytic and paralytic hosts. IDO expression was determined by real-time qRT-PCR using total RNA extracted from several tissues in mice showing a neurological disorder such as paralysis (paralytic) and mice showing no paralytic symptoms (aparalytic) 5 dpi. **c** Detection of IDO protein during JE progression. IDO protein levels in several tissues of JEV-infected mice were evaluated by Western blot using a monoclonal antibody specific for IDO. Differences in IDO protein levels between aparalytic and paralytic hosts were evaluated after mice were divided into groups showing either aparalytic (*AP*) or paralytic symptoms (*P*) 5 dpi. **d** IDO expression in primary myeloid cells, microglia, and cortical neurons after JEV infection. Bone marrow-derived DCs (*BMDCs*), plasmacytoid DCs (*pDCs*), macrophages (*BMDMs*), microglia, and primary cortical neurons were infected with JEV (10 MOI for BMDCs, pDCs, BMDMs, and microglia; 0.01 MOI for neurons) and IDO expression was determined by real-time qRT-PCR at the indicated time points. The levels of IDO expression were expressed as fold relative to mock-infected cells (0 h). *p < 0.05; **p < 0.01; ***p < 0.001 compared to levels in the indicated groups

To determine whether IDO expression changes during JE progression, several tissues obtained from JEV-infected BL/6 mice were used to evaluate IDO mRNA expression at the early stage of infection (from 0 to 3 dpi) prior to the presentation of neurological disorders. As shown in Fig. 1a, JEV infection induced no apparent changes in the expression of IDO in the examined tissues, including lymphoid (spleen, mesenteric LN, bone marrow), extraneural (liver), and CNS tissues (brain, spinal cord) prior to the onset of neurological disorders. In general, infected mice showed clinical signs starting with generalized piloerection, paresis, and rigidity, which then progressed to severe neurological signs, such as postural imbalance, ataxia, and generalized tonic-clonic seizure, by 4 to 5 dpi. Thus, we were interested in testing whether IDO expression varies between mice showing clinical signs, such as paralysis, and mice displaying no clinical signs. To this end, mice were divided into two populations showing either paralysis or no paralysis 5 dpi, the point at which approximately 30–40 % of infected mice showed neurological disorders, and the expression of IDO mRNA was evaluated in several tissues. Interestingly, enhanced induction of IDO expression was observed only in lymphoid tissues (spleen and bone marrow) and the CNS (brain and spinal cord) of mice showing paralysis compared to mice showing no paralysis (Fig. 1b). However, other extraneural tissues, including mesenteric LN and liver, showed no apparent increases in expression of IDO in paralyzed mice. IDO expression at the protein level was also confirmed by Western blotting using total lysates derived from several tissues. In agreement with the enhanced induction of IDO mRNA expression in paralyzed mice, mice showing paralysis displayed an apparent increase in the expression of IDO protein in lymphoid tissues (spleen, bone marrow) and neural tissues (brain and spinal cord) (Fig. 1c). DCs and macrophages in peripheral tissues are the primary target cells of JEV, and neuron cells support JEV replication after the virus gains access into the CNS. Also, microglia, tissue-resident macrophages in the CNS are believed to regulate neuroinflammation caused by various insults. Therefore, we evaluated IDO expression in primary myeloid-derived DCs, pDCs, macrophages, microglia, and primary cortical neuron cells after JEV infection. Conventional DCs (BMDCs), pDCs, macrophages (BMDMs), microglia, and neuron cells displayed different dynamic patterns of IDO expression, depending on the time after JEV infection (Fig. 1d). pDCs showed the most rapid expression of IDO, with levels peaking at 24 h pi, whereas BMDCs displayed a delayed expression pattern with the levels peaking at 48 h pi. IDO was also expressed in macrophages with gradual and moderate increases in levels up to 72 h pi, and microglia showed basal levels of IDO expression up to 24 h pi and increased levels thereafter. Neuron cells showed a gradual increase in IDO expression with approximately fivefold higher levels at 72 h pi compared to levels in the mock-infected group. This result indicates that the primary target cells in the periphery and CNS tissues can actively express IDO with different intrinsic patterns in response to JEV infection. Collectively, these results indicate that IDO expression in myeloid cells and neurons of the lymphoid and CNS tissues is closely associated with the clinical signs of JE. Notably, IDO expression in the neural tissues (brain and spinal cord) was likely to be coupled with neurological disorders such as paralysis.

### Blockage of IDO provides enhanced resistance to JE

**Fig. 2:**
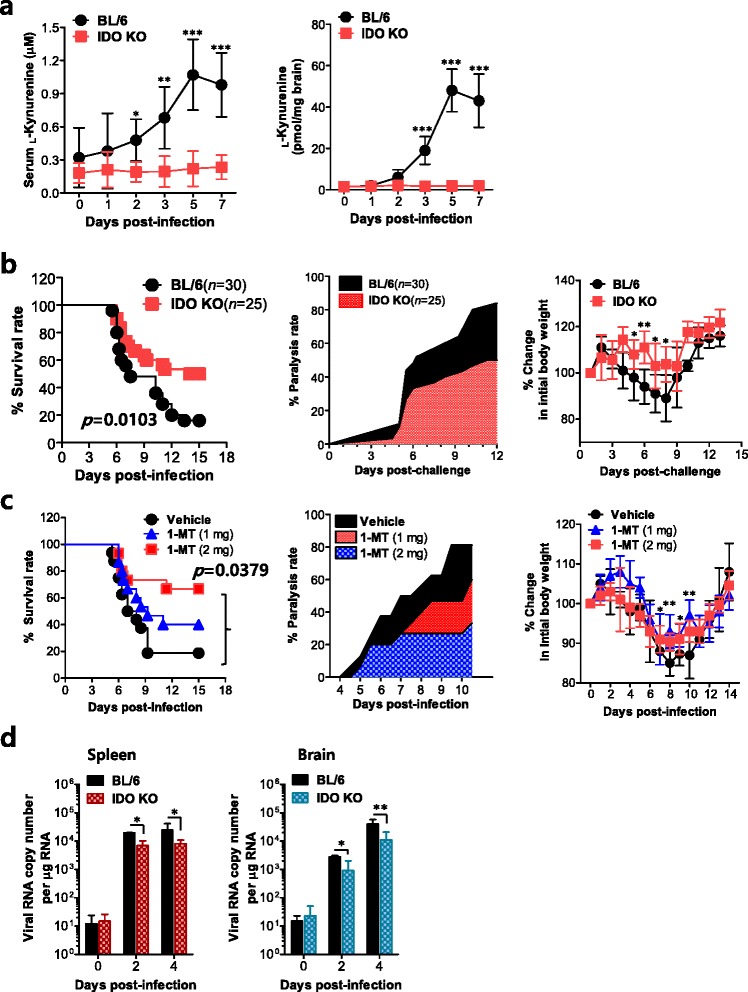
Blockage of IDO enhances resistance to JE and reduces viral burden. **a** Levels of l-kynurenine in the sera and brain of BL/6 and IDO KO mice. Following JEV infection, levels of l-kynurenine in the sera and brain homogenates were estimated by HPLC at different time points. **b** Susceptibility of IDO-ablated mice to JE. BL/6 and IDO KO mice (4 to 5 weeks old, *n* = 25–30) were inoculated i.p. with JEV (3.0 × 10^7^ PFU) and examined over 15 days for their survival. **c** Enhanced resistance to JE by IDO inhibition. BL/6 mice were infected i.p. with JEV and administered an IDO inhibitor (1-MT, 1 and 2 mg per mouse) every day. *Left graph*, curve showing survival rates; *middle graph*, proportion of mice showing neurological disorders during JE progression every 6 h from 4 to 11 dpi; *right graph*, changes in body weight. Changes in body weight were expressed as the mean percentage ± SD of body weight relative to the time of challenge. **d** Viral burden in lymphoid and inflammatory tissues during JE. Viral burden in the spleen and brain of infected mice was assessed by real-time qRT-PCR at the indicated time points post-infection. The viral RNA load was expressed as viral RNA copy number per microgram of total RNA (*n* = 5–7). *p < 0.05; **p < 0.01; ***p < 0.001 compared with levels in the indicated groups or in BL/6 control mice

Because IDO expression in lymphoid and neural tissues was closely associated with JE progression, we were interested in investigating the role of IDO during JE progression. BL/6 mice infected with JEV showed gradually elevated release of the tryptophan catabolite l-kynurenine by cells expressing functional IDO enzyme activity in the sera and brain homogenates relative to basal levels in IDO KO mice (Fig. 2a), which suggests that IDO expression increases during JE progression. Also, we examined and compared the susceptibility of IDO-deficient (KO) mice to JE caused by neurotropic JEV infection with that of wild-type BL/6 mice (Fig. 2b). The ablation of IDO resulted in a markedly increased survival rate of 55 % in IDO KO mice vs. 18 % in BL/6 mice after JEV infection (3.0 × 10^7^ PFU) (left curve in Fig. 2b), thereby signifying significantly enhanced resistance to JE. Likewise, IDO KO mice showed delayed signs of neurological disorder starting 5–6 dpi compared to BL/6 mice, which displayed signs of neurological disorder sooner and in a higher proportion of animals (middle graph in Fig. 2b), and IDO ablation resulted in less change in body weight (right graph in Fig. 2b). These results indicate that IDO ablation ameliorates JE progression. Furthermore, we tested the effect of the IDO-specific inhibitor 1-methyl-[d]-tryptophan (1-MT) on JE progression. As suggested by our other results, oral treatment with 1-MT resulted in reduced mortality, delayed neurological disorder, and less change in body weight compared to untreated BL/6 mice (Fig. 2c). Therefore, this result strengthens our finding that IDO ablation provides enhanced resistance to JE. To better understand JE progression in IDO-ablated mice, we examined viral burden in peripheral lymphoid and CNS tissues after JEV infection. IDO KO mice were observed to contain less amount of virus, with around a tenfold decrease in the spleen and brain compared to levels in BL/6 mice (Fig. 2d). Ultimately, these results suggest that IDO ablation ameliorates JE progression by regulating the viral burden in peripheral lymphoid and CNS tissues.

### Blockage of IDO results in early and increased CNS infiltration by myeloid and lymphoid leukocytes

**Fig. 3:**
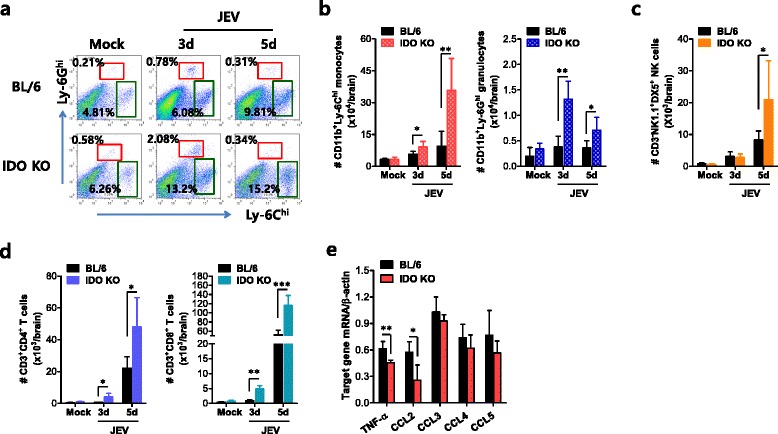
IDO ablation results in early and increased CNS infiltration by myeloid and lymphoid leukocytes. **a**, **b** The frequency and number of Ly-6C^hi^ monocytes and Ly-6G^hi^ granulocytes in the CNS. The frequency (**a**) and total number (**b**) of Ly-6C^hi^ monocytes and Ly-6G^hi^ granulocytes in the CNS were determined by flow cytometric analyses 3 and 5 dpi using vigorous heart perfusion. Values presented in the representative dot plots denote the average percentage of the indicated population after gating on CD11b^+^ cells (*n* = 4–5). **c**, **d** Accumulated number of NK cells, CD4^+^, and CD8^+^ T-cells in the CNS. Total accumulated number of NK cells (CD3^−^NK1.1^+^DX5^+^), CD4^+^ (CD3^+^CD4^+^), and CD8^+^ (CD3^+^CD8^+^) T-cells in the CNS were enumerated by flow cytometric analysis 3 and 5 dpi. **e** The expression of TNF-α and CC chemokines in the CNS. The expression levels of TNF-α and CC chemokines were determined by real-time qRT-PCR using total RNA extracted from brain tissue 2 dpi. Data show the average ± SD of the indicated cell populations derived from at least five mice per group. *p < 0.05; **p < 0.01; ***p < 0.001 compared with levels in the indicated groups

CNS infiltration by CD11b^+^Ly-6C^hi^ monocytes is a hallmark of CNS inflammation caused by neurotropic viral infection [29]. These cells migrate into the infected brain, where they differentiate into DC, macrophage, and microglia populations [30–33]. Although the potential contribution of CD11b^+^Ly-6C^hi^ monocytes to neuroinflammation remains controversial, CNS infiltration by CD11b^+^Ly-6C^hi^ monocytes is believed to support their protective role during lethal neuroinflammation [34–37]. Therefore, in order to better understand the amelioration of JE in IDO KO mice, we examined CNS infiltration by leukocytes, including CD11b^+^Ly-6C^hi^ monocytes, during JE progression. Wild-type BL/6 and IDO KO mice contained comparable levels of CD11b^+^Ly-6C^hi^ monocytes and CD11b^+^Ly-6G^hi^ granulocytes in the brain before JEV infection. However, the frequency of CD11b^+^Ly-6C^hi^ monocytes gradually increased in the CNS of IDO KO mice during JE progression (Fig. 3a). Also, CNS infiltration by CD11b^+^Ly-6G^hi^ granulocytes was transiently increased in IDO KO mice 3 dpi, after which the Ly-6G^hi^ granulocyte frequency was comparable in both BL/6 and IDO KO mice 5 dpi. Similarly, the accumulated total number of CNS-infiltrated Ly-6C^hi^ monocytes and Ly-6G^hi^ granulocytes was increased in IDO KO mice, compared to those of BL/6 mice (Fig. 3b). Furthermore, because CD4^+^ Th1, CD8^+^, and NK cells may play beneficial roles in controlling JE progression [38–41], we examined CNS infiltration of NK, CD4^+^, and CD8^+^ T-cells. As with CNS infiltration by Ly-6C^hi^ monocytes, CNS infiltration by NK, CD4^+^, and CD8^+^ T-cells was observed to gradually increase in IDO KO mice compared to levels observed in BL/6 mice (Fig. 3c, d). Notably, CD8^+^ T-cells showed marked infiltration with threefold increased levels in the CNS of IDO KO mice. With respect to CNS inflammation, the expression of cytokines and chemokines within the CNS may be required to fully explain encephalitis, because encephalitis caused by neurotropic viruses is indirectly derived from CNS degeneration caused by robust immunological responses, such as the uncontrolled secretion of cytokines, including TNF-α, and the resultant activation of microglia and astrocytes [22, 23]. Therefore, we examined the expression of TNF-α and CC chemokines in the CNS. Our results revealed that the expression of CC chemokines was comparable in both BL/6 and IDO KO mice, except that the expression levels of TNF-α and CCL2 were modestly decreased in IDO KO mice (Fig. 3e). This result implies that CC chemokine expression may not play a role in increased CNS infiltration by Ly-6C^hi^ monocytes, NK, CD4^+^, and CD8^+^ T-cells. Collectively, these results suggest that IDO ablation allows for early and increased CNS infiltration by myeloid and lymphoid leukocytes, which may mediate the early control of viral replication in the CNS.

### IDO ablation enhances the activation of NK cells during JE

**Fig. 4:**
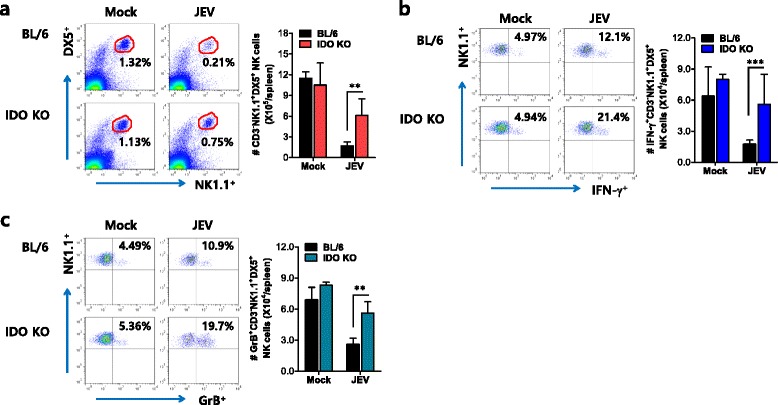
Enhancement of NK cell activation by IDO ablation. **a** NK cell frequency and number. The frequency and absolute number of CD3^−^NK1.1^+^DX5^+^ NK cells in the spleen were determined by flow cytometric analysis 2 dpi. Values in representative dot plots on the left denote the average percentage of splenic NK cells derived from at least four mice per group, after gating on CD3^−^ cells; the *bar graph on the right* denotes the average ± SD of splenic NK cell numbers derived from at least four mice per group. **b**, **c** Activation of NK cells. The activation of CD3^−^NK1.1^+^DX5^+^ NK cells was evaluated by intracellular IFN-γ and granzyme B (GrB) staining upon stimulation with PMA plus ionomycin. Values in the representative dot plots on the left denote the average percentage of IFN-γ- or GrB-producing NK cells derived from at least four mice per group; the *bar graphs on the right* show the average number ± SD of NK cells producing IFN-γ or GrB in the spleens of at least four mice per group. **p < 0.01; ***p < 0.001 compared with levels in the indicated groups

Antiviral innate NK cell activation is believed to play an important role in regulating JE progression through the control and clearance of JEV in extraneural and neural tissues [38]. Therefore, in order to characterize the immunological parameters associated with control of JEV replication in IDO KO mice, we examined and compared NK cell responses in both BL/6 and IDO KO mice. Because JEV was administered intraperitoneally, analysis of the spleen provides insight into how IDO modulates the innate immune and inflammatory responses during the early phase of infection. Analysis of splenic NK cells revealed that BL/6 mice exhibited a reduction in CD3^−^NK1.1^+^DX5^+^ NK cells following JEV infection (Fig. 4a), as if wild-type mice previously showed decreased number of NK cells [24]. However, IDO KO mice showed less of a reduction in the number of CD3^−^NK1.1^+^DX5^+^ NK cells. Consequently, an increase in the total number of splenic NK cells was detected in IDO KO mice compared to BL/6 mice. Moreover, when the activation of NK cells was evaluated by the production of IFN-γ and granzyme B from NK cells, the frequency and the total number of CD3^−^NK1.1^+^DX5^+^ NK cells producing IFN-γ and granyzme B were apparently increased in IDO KO mice (Fig. 4b, c). Therefore, this result indicates that increased activation of NK cells plays a role in the early control of JEV replication, thereby resulting in the amelioration of JE progression.

### Enhancement of JEV-specific CD4^+^ and CD8^+^ T-cell responses by IDO ablation

**Fig. 5:**
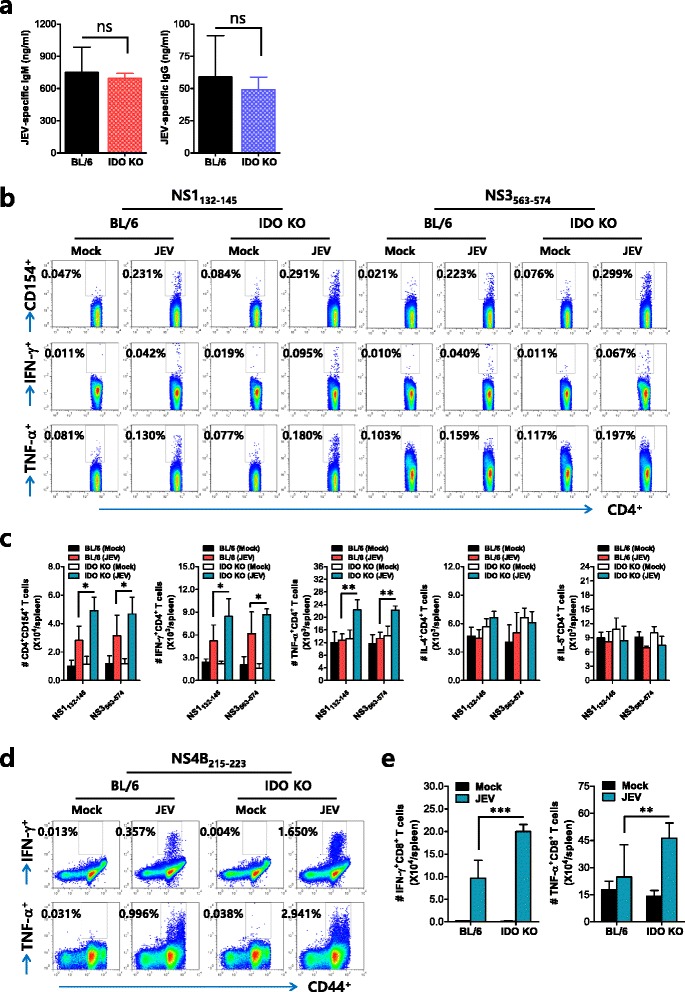
IDO ablation enhances CD4^+^ and CD8^+^ T-cell responses specific for JEV antigen. **a** JEV E-specific IgM and IgG response. Sera were collected from surviving mice 7 dpi and used in ELISA to detect IgM and IgG levels specific for the JEV E protein. The data show the average ± SD of the JEV E-specific IgM and IgG levels derived from surviving mice (*n* = 6–8). **b**, **c** CD4^+^ T-cell response specific for JEV antigen. The splenocytes prepared from surviving mice (*n* = 4–5) were stimulated with the JEV epitope peptides of CD4^+^ T-cells (NS1_132–145_ and NS3_563–574_) for 12 h. The frequency (**b**) and absolute number (**c**) of CD4^+^ T-cells specific for the JEV epitope peptides were determined by intracellular CD154, IFN-γ, or TNF-α staining, combined with surface CD4 staining. **d**, **e** CD8^+^ T-cell response specific for JEV antigen. The frequency (**d**) and absolute number (**e**) of CD8^+^ T-cells specific for the JEV epitope peptide (NS4B_215–223_) were determined by intracellular IFN-γ or TNF-α staining after an 8-h stimulation with peptide. Values in the representative dot plots denote the average percentage of the indicated cell population, and the *bar charts* show the average ± SD of the values derived from at least four mice per group. *p < 0.05; **p < 0.01; ***p < 0.001 compared with levels in the indicated groups

In addition to NK cells, adaptive immune responses specific for JEV antigen are required for the regulation of JE progression through peripheral control of JEV replication [38–41]. Furthermore, IDO may in some cases affect antigen-specific antibody responses [42, 43], which contribute to the control of JEV dissemination and replication in the brain. Our data revealed that IDO ablation induced no significant changes in serum IgM and IgG specific for JEV antigen (Fig. 5a). Because IDO is known to suppress T-cell-mediated adaptive immune responses in a range of clinically relevant syndromes, including autoimmune, allergic, and infectious diseases [9, 10], we also evaluated CD4^+^ and CD8^+^ T-cell responses specific for JEV antigen in surviving BL/6 and IDO-ablated mice 7 dpi. IDO ablation resulted in moderately increased JEV-specific CD4^+^ T-cell responses when CD4^+^ T-cell responses were evaluated by intracellular CD154, IFN-γ, and TNF-α staining in response to stimulation with two epitope-peptides (NS1_132–145_ and NS3_563–574_) derived from JEV (Fig. 5b). Consistent with this finding, the total number of CD4^+^ T-cells producing IFN-γ or TNF-α in response to JEV epitope stimulation was higher in IDO-ablated mice (Fig. 5c). Furthermore, somewhat interestingly, IDO-ablated mice displayed markedly increased CD8^+^ T-cell responses with three- to fivefold higher levels, compared to BL/6 mice (Fig. 5d), and consistently contained a higher total number of JEV-specific CD8^+^ T-cells producing IFN-γ or TNF-α in response to a JEV CD8^+^ T-cell epitope (NS4B_215–223_) (Fig. 5e). These results indicate that the increased CD4^+^ and CD8^+^ T-cell responses generated in IDO-ablated mice could contribute to the control of JE progression during the late stage of infection.

### IDO ablation induces a skewed IFN-γ^+^CD4^+^ Th1 response during JE

**Fig. 6:**
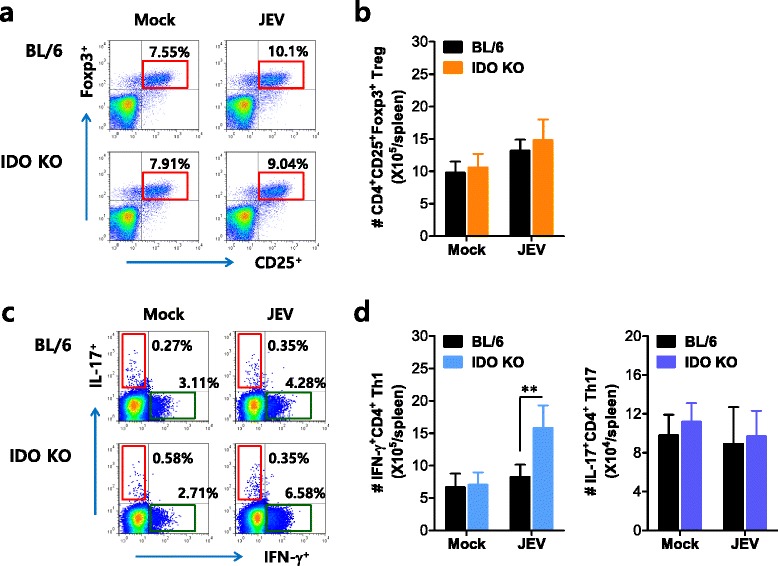
IDO induces a skewed IFN-γ^+^CD4^+^ T-cell response during JE. **a**, **b** The frequency and number of CD4^+^Foxp3^+^ Tregs in the spleen of IDO-ablated mice. The frequency (**a**) and absolute number (**b**) of CD4^+^Foxp3^+^ Treg cells in the spleen of wild-type and IDO KO mice were determined by flow cytometric analysis 5 dpi. The values in the representative dot plots show the average percentage of CD25^+^Foxp3^+^ cells in CD4^+^ T-cells; the *bar charts* denote the average number ± SD of CD4^+^Foxp3^+^ Tregs in the spleen derived from at least four mice per group. **c**, **d** The frequency and number of IFN-γ^+^CD4^+^ Th1 and IL-17^+^CD4^+^ Th17 cells in the spleen of IDO-ablated mice. The frequency and number of IFN-γ^+^CD4^+^ Th1 and IL-17^+^CD4^+^ Th17 cells were determined by intracellular cytokine staining of the splenocytes prepared from wild-type and IDO KO mice 5 dpi in response to PMA + ionomycin stimulation. The values in the representative dot plots show the average percentage of the indicated cell populations after gating on CD4^+^ T-cells; the *bar charts* denote the average number ± SD of IFN-γ^+^CD4^+^ Th1 and IL-17^+^CD4^+^ Th17 cells in the spleen derived from at least four mice per group. **p < 0.01 compared with levels in the indicated groups

CD4^+^CD25^+^Foxp3^+^ Treg cells may contribute to controlling lethal neuroinflammation caused by neurotropic viruses [44]. In contrast, IL-17^+^CD4^+^ Th17 cells play a critical role in autoimmune and virus-caused immunopathologic diseases by facilitating pathologic consequences through neutrophil recruitment [45, 46]. The generation of CD4^+^CD25^+^Foxp3^+^ Tregs is reciprocally linked with IL-17^+^CD4^+^ Th17 and IFN-γ^+^CD4^+^ Th1 cells [47, 48]. Because IDO may regulate the equilibrium of CD4^+^Foxp3^+^ Tregs and IL-17^+^CD4^+^ Th17 cells in inflammatory diseases [49, 50], we examined the generation of each CD4^+^ Th subset, including CD4^+^Foxp3^+^ Tregs, IL-17^+^CD4^+^ Th17, and IFN-γ^+^CD4^+^ Th1 cells, early during JE progression. Our results revealed that IDO-ablated mice showed no significant alterations in the frequency or number of CD4^+^CD25^+^Foxp3^+^ Tregs, compared to wild-type BL/6 mice (Fig. 6a, b). In contrast, IFN-γ^+^CD4^+^ Th1 cells were detected at higher levels in IDO-ablated mice, and the frequency and number of IL-17^+^CD4^+^ Th17 cells were not changed by IDO ablation (Fig. 6c, d). Therefore, this result suggests that the increase in IFN-γ^+^CD4^+^ Th1 cells in IDO-ablated mice contributes in part to the control of JE progression during the early stage of infection.

### IDO ablation enhances innate IFN-I responses of CD11c^+^ DCs following JEV infection

**Fig. 7:**
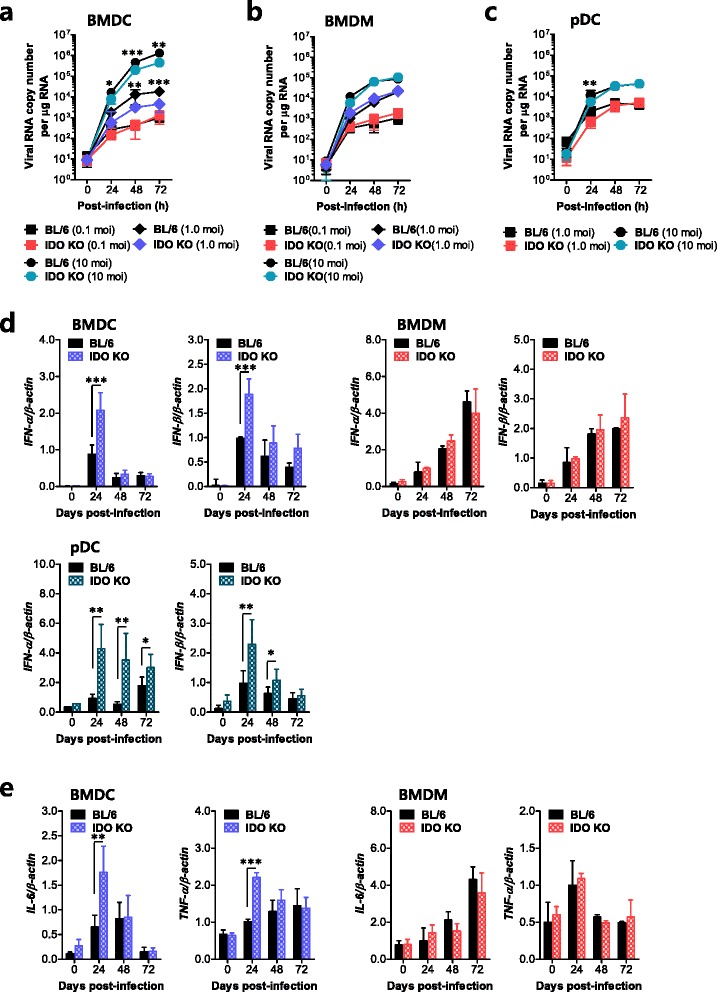
IFN-I innate immune responses of IDO-ablated myeloid-derived cells after JEV infection. Primary bone marrow-derived conventional DCs (BMDCs), plasmacytoid DCs (pDCs), and macrophages (BMDMs) recovered from BL/6 and IDO KO mice were infected with JEV at MOIs of 0.1, 1.0, and 10 for viral replication and 10 for cytokine expression. JEV replication and the expression of cytokines and IFN-α/β were evaluated by real-time qRT-PCR using extracted total RNA. **a**–**c** JEV replication in BMDCs, BMDMs, and pDCs. **d**, **e** IFN-α/β, IL-6, and TNF-α expression in BMDCs, pDCs, and BMDMs. The *bar charts* show the average ± SD of the values derived from BMDCs, BMDMs, and pDCs assayed in quadruplicates. *p < 0.05; **p < 0.01; *p* < 0.001 compared with levels in the indicated groups

**Fig. 8:**
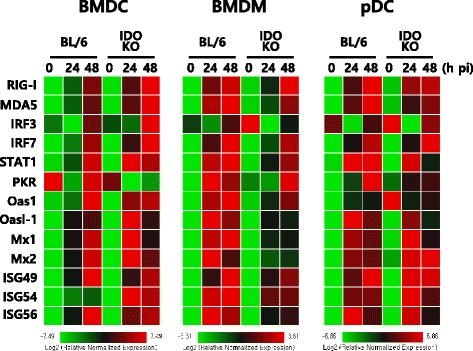
The expression of RLR, IRF, and ISG genes in BMDCs, BMDMs, and pDCs following JEV infection. BMDCs, BMDMs, and pDCs recovered from WT and IDO KO mice were infected with JEV at a MOI of 10 and used to analyze the induction of IRF, ISG, and RLR genes at 24 and 48 h pi. The expression of each IRF, ISG, and RLR gene was normalized to that of β-actin after determining the mRNA levels by real-time qRT-PCR and displayed as the average of at least four independent samples, according to the indicated color on a log_2_ scale

Myeloid cells, including DCs and macrophages, are the primary target cells of JEV infection in the peripheral tissues and function to regulate the spread of virus to distant tissues such as the CNS [23]. Furthermore, myeloid cells can produce IFN-I via PRR recognition upon JEV infection, which plays a crucial role in controlling viral replication [51, 52]. Additionally, IDO ablation appears to regulate viral replication via IFN-I production [21]. Because viral loads at the periphery in IDO-ablated mice were lower than in wild-type BL/6 mice, we evaluated the contribution of DC subsets (conventional and plasmacytoid) and macrophages to the IFN-I innate immune responses caused by JEV infection in the IDO-ablated environment. In order to assess the role of IDO in inducing the IFN-I innate response of JEV-infected DC subsets and macrophages, BMDCs, pDCs, and macrophages (BMDMs) prepared from IDO-ablated mice were infected with JEV and used to evaluate viral replication and the induction of IFN-I and pro-inflammatory cytokines. Interestingly, IDO-ablated BMDCs and pDCs, but not BMDMs, showed significantly lower JEV replication (Fig. 7a–c). Notably, BMDCs derived from IDO-ablated mice exhibited impaired JEV replication throughout the examination period compared to replication in cells from wild-type BL/6 mice. Furthermore, the inhibition of JEV replication in BMDCs and pDCs derived from IDO-ablated mice was closely associated with enhanced expression of IFN-I (IFN-α/β) following JEV infection (Fig. 7d). IDO-ablated BMDCs and pDCs, but not BMDMs, showed rapid induction of IFN-α/β in response to JEV infection compared to levels measured in cells from wild-type BL/6 mice. However, it was curious that pDCs showed less apparent regulation of viral replication than BMDCs, despite pDCs inducing stronger IFN-I innate responses. Presumably, this discrepancy is related to cell-intrinsic properties or other mechanisms involved in regulating viral replication. In addition, rapid and increased induction of IL-6 and TNF-α mRNAs was observed upon JEV infection in IDO-ablated BMDCs (Fig. 7e). Collectively, these results imply that rapid and increased IFN-I innate responses in CD11c^+^ DC subsets (conventional DCs and pDCs) may contribute to the early control of viral replication in the absence of IDO. To further characterize IFN-I innate responses in JEV-infected DC subsets derived from IDO-ablated mice, we also measured the induction levels of ISG genes. We specifically focused on pattern recognition receptors ( PRRs; RIG-I [DDX1], MDA5 [IFITH1], PKR), transcription factors (STAT1, IRF3, IRF7), IFN-induced antivirus-related genes (Oas1, Oasl-1, Mx1, Mx2), and several ISG genes (ISG49 [IFIT3], ISG54 [IFIT2], ISG56 [IFIT1]). Our results revealed that BMDCs and pDCs derived from IDO-ablated mice showed rapid and enhanced expression of the STAT1, Oas1, Mx1, Mx2, and ISG genes with slightly different patterns at 24 h pi, whereas BMDMs derived from IDO-ablated mice showed either no alteration or a slight decrease in the expression of PRRs, IFR3 and IRF7, and ISG genes (Fig. 8). Also, it was interesting that BMDCs and pDCs derived from IDO-ablated mice displayed early enhanced induction of PRRs (RIG-I and MDA5) and their transcription factor (IRF7) at 24 h pi. This indicates that rapid and increased expression of ISG genes in JEV-infected BMDCs and pDCs follows the inhibition of JEV replication via enhanced expression of IFN-α/β. Collectively, IDO ablation appears to provide rapid and increased responses of IFN-I innate immunity in myeloid-derived DCs and pDCs upon JEV infection, thereby contributing to the amelioration of JE through early control of viral replication.

### Viral replication and innate immune responses of IDO-ablated microglia in response to JEV infection

**Fig. 9:**
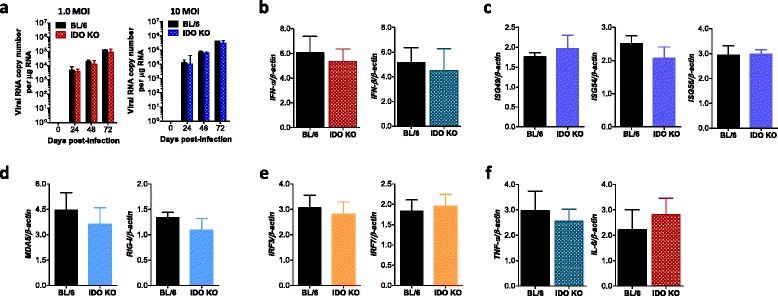
Viral replication and innate immune responses of IDO-ablated microglia in response to JEV infection. Primary microglia recovered from BL/6 and IDO KO mice were infected with JEV at MOIs of 1.0 and 10 for viral replication and 10 for cytokine expression. JEV replication and the expression of cytokines, IFN-α/β, RLRs, ISGs, and IRF transcription factors were evaluated by real-time qRT-PCR using extracted total RNA at the indicated time point or 24 h pi. **a** JEV replication. **b**, **c** The expression of IFN-α/β and the ISG gene. **d**, **e** RLR and IRF expression. **f** TNF-α and IL-6 expression. The *bar charts* show the average ± SD of values derived from primary microglia assayed in quadruplicates

Microglia cells are CNS-resident macrophages that play an important role in regulating the neuroinflammation caused by sterile and non-sterile insults [53]. Bystander damage caused by pro-inflammatory mediators released from microglia likely contributes to the exacerbation of JE progression. Furthermore, because IDO-ablated microglia are considered to show stronger inflammatory responses after the virus gains access to the CNS, we examined the innate immune responses of primary microglia cells derived from the brain of fetal BL/6 and IDO KO mice in response to JEV infection. Our results revealed that primary microglia recovered from the brain of IDO-ablated mice showed similar innate immune responses to JEV infection as shown in macrophages derived from BM cells of IDO KO mice. IDO-ablated microglia were observed to allow JEV replication with moderately but not significantly lower levels compared to those of BL/6 mice (Fig. 9a). Consistent with this, IDO-ablated microglia displayed no apparent enhancement of IFN-I innate responses when the expression of IFN-α/β and ISG genes (ISG49, ISG54, ISG56) was examined (Fig. 9b, c). Also, no significant differences in the expression of RLRs and transcription factors IRF3 and IRF7 were observed between microglia derived from IDO KO and BL/6 mice (Fig. 9d, e), and both IDO-ablated and wild-type microglia showed comparable expression of pro-inflammatory cytokines TNF-α and IL-6 in response to JEV infection (Fig. 9f). Therefore, these results indicate that microglia could not provide dominant contribution to the regulation of JEV dissemination and JE progression in the CNS under an IDO-ablated environment.

### Induction of IFN-I and ISG in primary cortical neurons derived from IDO KO mice after JEV infection

**Fig. 10:**
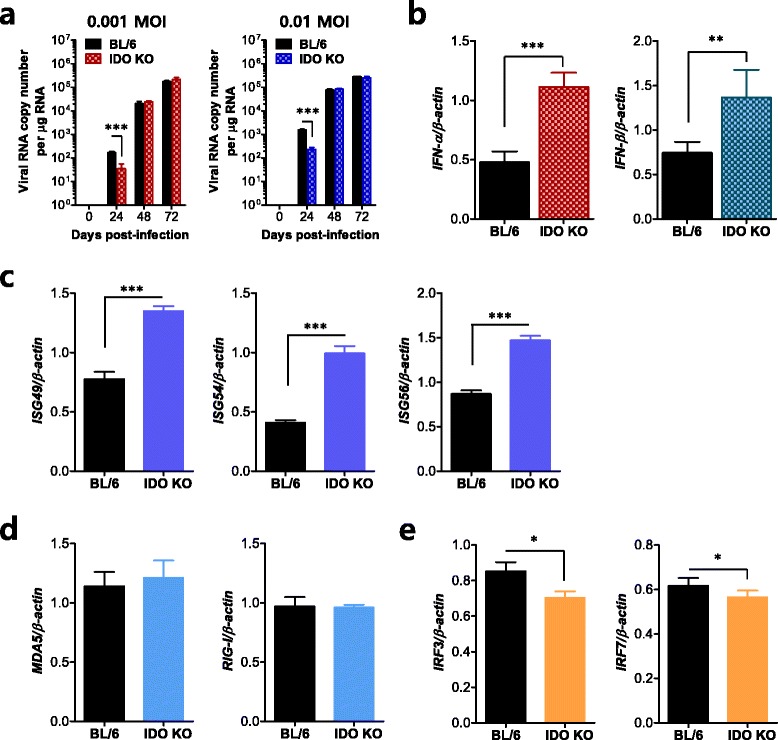
Induction of IFN-I and ISGs in primary cortical neurons derived from IDO KO mice after JEV infection. Primary cortical neurons generated from BL/6 and IDO KO mice were infected at MOIs of 0.001 and 0.01, and viral replication and IFN-I responses 24, 48, and 72 h pi were analyzed by real-time qRT-PCR. **a** JEV replication. **b** IFN-I (IFN-α/β) expression. **c**–**e** Induction of ISGs, RLRs, and IRFs in infected primary cortical neuron. The expression of IFN-I (**b**), ISGs (**c**), RLRs (**d**), and IRFs (**e**) was determined using primary cortical neuron infected with JEV (0.1 MOI) 24 h pi. The *bar charts* show the average ± SD of values derived from primary cortical neurons in quadruplicate. *p < 0.05; **p < 0.01; ***p < 0.001 compared with levels in the indicated groups

Neurons are the main target cell of JEV replication within the CNS, and their death is a key factor in the pathogenesis and neurological sequelae of JEV [23]. Furthermore, neuron cells have also been shown to produce antiviral IFN-I in response to viral infection and are consequently involved in controlling viral replication in the CNS [54, 55]. Therefore, we examined viral replication and IFN-I innate immune responses in primary cortical neuron cells generated from wild-type BL/6 and IDO-ablated mice after JEV infection. Consistent with the results obtained from BMDCs and pDCs, primary cortical neurons derived from IDO-ablated mice showed transiently reduced JEV replication (Fig. 10a). This transient reduction of JEV replication in IDO-ablated neurons was associated with significantly increased induction of IFN-I (IFN-α/β), relative to the levels measured in cells from wild-type BL/6 mice (Fig. 10b). Also, it seemed that the expression of antiviral ISG genes (ISG49, ISG54, ISG59) in IDO-ablated neurons followed IFN-I innate responses and the transient reduction in viral replication (Fig. 10c), whereas the expression levels of transcription factors (IRF3, IRF7), but not PRRs (RIG-I, MDA5), were decreased by JEV infection (Fig. 10d, e). Therefore, these results suggest that the ablation of IDO could provide enhanced IFN-I innate immune responses in neuron cells to regulate the spread of JEV in the CNS.

### IDO ablation in HSC-derived leukocytes alleviates JE via enhancement of the IFN-I innate response

**Fig. 11:**
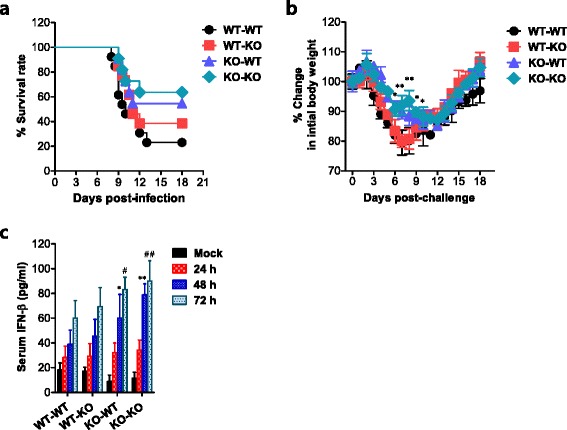
IDO ablation in HSC-derived leukocytes alleviates JE via enhancement of the IFN-I innate response. BM cells from WT or IDO KO mice were grafted to lethally irradiated WT or IDO KO recipient mice, which were then infected with JEV (3.0 × 10^7^ PFU). **a** Susceptibility of IDO KO BM chimeric models to JE. Infected recipient mice (*n* = 12) were examined over 18 days to determine the survival rate. **b** Changes in body weight. Data are expressed as the average percentage of body weight relative to the time of challenge. **c** Systemic IFN-β levels in an IDO KO BM chimera. The amount of serum IFN-β was determined by ELISA at the indicated time points. *p < 0.05; **p < 0.01 compared with levels in WT-WT BM chimera 48 h pi. #*p* < 0.05; ##*p* < 0.01 compared with levels in WT-WT BM chimera 72 h pi

Our results support the idea that IDO ablation ameliorates JE progression by provoking potent antiviral IFN-I innate responses in myeloid-derived DCs and pDCs at the periphery. Therefore, we were interested in confirming whether myeloid cells derived from hematopoietic stem cells (HSCs) play a dominant role in regulating JE progression in IDO-ablated mice via the induction of enhanced IFN-I innate responses. To this end, we used a BM chimeric model of wild-type BL/6 and IDO-ablated mice. In support of our hypothesis, our results revealed that myeloid cells derived from HSCs played a dominant role in conferring amelioration of JE in the IDO-ablated environment. Specifically, wild-type BL/6 recipients of IDO KO BM donor cells (KO-WT) showed enhanced resistance to JE, compared to the IDO KO recipients of wild-type BL/6 BM donor cells (WT-KO) and wild-type BL/6 recipients of wild-type BL/6 BM donor cells (WT-WT) (Fig. 11a). Furthermore, KO-WT and KO-KO BM chimeric models showed less change in body weight after JEV infection compared to the other BM chimeric models (Fig. 11b). Also, potent and rapid IFN-I innate immune responses were observed in KO-WT and KO-KO BM chimeric models compared to those observed in the WT-WT BM chimeric model, as evaluated by the determination of serum IFN-β (Fig. 11c). This result indicates that myeloid cells derived from HSCs of IDO-ablated mice play a dominant role in the IFN-I innate response in IDO KO hosts. Collectively, it appears that the ablation of IDO in myeloid cells derived from HSCs has an important role in ameliorating JE by inducing a potent and rapid IFN-I innate immune response.

## Discussion

In this study, IDO expression from myeloid and neuron cells located in extraneural and neural tissues during JE progression appears to be closely associated with clinical signs such as neurological disorders. Furthermore, we demonstrated that inhibition of IDO activity through genetic ablation or use of an IDO inhibitor (1-MT) enhanced the resistance to JE progression induced by JEV infection. In our mechanistic studies on the role of IDO in regulating JE progression, IDO-ablated mice showed early and increased CNS infiltration by myeloid cells (Ly-6C^hi^ monocytes and Ly-6G^hi^ granulocytes) and lymphoid cells (CD3^−^NK1.1^+^DX5^+^ NK, CD4^+^, and CD8^+^ T-cells), which were associated with a reduced viral burden in the CNS. IDO ablation increased the activity of cytolytic effector cells following JEV infection, due to the enhancement of IFN-γ or granzyme B-producing NK cells and JEV-specific CD8^+^ T-cell responses in IDO-ablated mice. In addition, the enhanced production of IFN-γ from CD4^+^ Th1 cells in response to JEV antigen might contribute to early viral clearance from extraneural and neural tissues of IDO-ablated mice. Also, somewhat intriguingly, our data revealed that potent and rapid IFN-I innate immune responses in CD11c^+^ DCs and neuron cells following JEV infection could effectively regulate the spread of JEV in IDO-ablated mice. This finding was supported by the results that IDO ablation in myeloid cells derived from HSCs played a dominant role in ameliorating JE and that IDO-ablated HSC-derived leukocytes were major contributors to IFN-I innate responses in the host. Collectively, our data suggest that the ablation of IDO activity with specific inhibitors, such as 1-MT, could be a valuable therapeutic tool for the treatment of JE.

IDO is considered a negative regulator of the immune system because IDO activity in APCs, including DCs and macrophages, is a potent mechanism for inducing immunosuppression and tolerance. Therefore, it is probable that the attenuation of immunosuppressive IDO activity leads to enhanced innate and adaptive immune responses for viral clearance. Indeed, the inhibition of IDO activity with a specific inhibitor resulted in enhanced CD4^+^ Th1-type and CD8^+^ T-cell responses specific to the influenza virus [20]. Furthermore, the inhibition of IDO activity reportedly modifies the immunodominance hierarchy by increasing the number of CD8^+^ T-cells specific to a subdominant epitope derived from the influenza virus. The ablation of IDO also suppresses viral replication in retrovirus-infected mice through IFN-I production [21]. These findings strongly support our data that IDO ablation ameliorates JE progression through enhanced type I/II IFN innate and adaptive immune responses. Although the in vivo role of IDO after viral infection has been investigated in a few studies using murine infection models, the regulatory role of IDO in neuroinflammation caused by neurotropic viruses, such as JEV, has not been addressed to date. To the best of our knowledge, our findings provide the first evidence that IDO inhibition could regulate the in vivo progression of viral encephalitis caused by neurotropic viruses such as JEV and WNV.

The molecular pathogenesis of JE remains unclear, but JE is considered an immunopathological disease since uncontrolled over-activation of microglia/glia and infiltrated leukocytes after CNS invasion of JEV drives neurological disorders [22, 23]. Although CNS infiltration by peripheral innate and adaptive immune cells appears to play a critical role in host defense against infections with neurotropic viruses such as JEV, control of CNS infiltration is also required because excessive or inappropriate CNS infiltration can cause profound damage. Therefore, the outcome of JE pathogenesis appears to depend on the complicated interplay between virus-induced tissue damage and host immune response against JEV antigens. CNS infiltration by CD11b^+^Ly-6C^hi^ monocytes is a hallmark of neuroinflammation caused by neurotropic viruses. These CD11b^+^Ly-6C^hi^ monocytes migrate into the inflamed CNS, where they differentiate into DCs, macrophages, and microglia to regulate neuroinflammation [30–37]. Although conflicting roles have been postulated for Ly-6C^hi^ monocytes in CNS inflammation, CNS infiltration by CD11b^+^Ly-6C^hi^ monocytes is essential in the control of neuroinflammation caused by neurotropic viruses, which supports their protective role during CNS inflammation [34–37]. Notably, the differentiation state of Ly-6C^hi^ monocytes that infiltrate into the CNS appears to affect the progression of neuroinflammation caused by various insults [56–58]. Supportively, IDO ablation was observed to lead to enhanced CNS infiltration of CD11b^+^Ly-6C^hi^ monocytes, which may contribute to a beneficial outcome in JE. Furthermore, early and increased infiltration of NK, CD4^+^, and CD8^+^ T-cells in the CNS of IDO-ablated mice appears to play a role in reducing the viral burden, because infiltration of NK, CD4^+^ Th1, and CD8^+^ T-cells producing IFN-γ is required for early clearance of the virus from the CNS [38–41]. After peripheral introduction of JEV via mosquito bites, JEV initially replicates in its primary target cells, including DCs and macrophages, at the periphery and subsequently gains entry into the CNS through the BBB. Presumably, JEV-specific CD4^+^ Th1 and CD8^+^ T-cell responses generated in the peripheral lymphoid tissues of IDO-ablated mice would likely provide more effective control of viral replication, thereby reducing the amount of virus supplied from the periphery. In support of this notion, our data revealed that the viral burden in the extraneural and neural tissues of IDO-ablated mice were lower than those in wild-type BL/6 mice. The depletion or adoptive transfer of specific cell populations of NK and CD8^+^ T-cells was previously reported to provide no significant contribution to host survival and viral clearance [38]. Furthermore, co-transfer of immune CD4^+^ and CD8^+^ T-cells, but not individual transfer of either T-cell subpopulation, significantly contributed to a favorable JE outcome and promoted host survival [40]. These facts suggest the possibility that enhancement of broad immunity, including NK, CD4^+^ Th1, and cytotoxic CD8^+^ T-cells, in IDO-ablated mice accounts for the effective regulation of early viral clearance in extraneural and neural tissues, thereby providing protection against JE progression without tissue injury. However, IDO ablation did not result in any significant change in the humoral immune responses specific for JEV antigen. The effects of IDO on the humoral immune response appear to be variable, depending on the experimental model used [42, 43]. Because B-cell-deficient (μMT) mice uniformly succumbed to viral encephalitis caused by flaviviruses in a previous study, one obvious conclusion is that an early and sustained humoral immune response is absolutely essential to surviving infection with JEV and other related flaviviruses [59]. This notion suggests a limitation that IDO inhibition may not provide the perfect protection against JE progression due to the lack of substantial enhancement in the humoral response against JEV antigen. Thus, further studies on how to enhance the humoral immune response are needed in the context of IDO inhibition.

The requirement of IFN-γ in the recovery from infections with flaviviruses has been shown to be variable. IFN-γ provides an early protective immune response against a virulent North American isolate of WNV [60] and mouse-adapted strains of dengue virus [61, 62], whereas IFN-γ is dispensable in the control of infection with less virulent strains of WNV [63] or of yellow fever virus [64, 65]. Additionally, IFN-γ shows only a modest protective role against Murray Valley encephalitis [66]. Similarly, in the JE model, IL-12-mediated induction of IFN-γ has been reported to result in suppressed protective immunity [67], whereas IFN-γ was associated with a beneficial effect on the outcome of JE [38]. Our data favor the latter result, showing a beneficial role of IFN-γ in JE progression, because IDO ablation markedly increased IFN-γ-producing NK, CD4^+^ Th1, and CD8^+^ T-cells. IFN-γ is believed to play diverse roles in infectious diseases, including the activation and polarization of CD4^+^ Th cells, upregulation of Fas in infected target cells, upregulation of MHC I and II-restricted Ag-presentation pathways, macrophage activation, and direct antiviral activity that overlaps with activities triggered by IFN-I [68]. Also, IFN-γ produced from CD4^+^ Th1 and CD8^+^ T-cells in IDO-ablated mice is believed to induce the maturation of Ly-6C^hi^ monocytes, which may contribute to better JE outcomes [69, 70]. However, IFN-γ-producing NK cells do not appear to significantly contribute to host survival, because NK-cell-depleted mice showed no change in viral burden or survival [38]. Furthermore, flaviviruses including WNV exhibit immune escape from NK cell attack via the upregulation of MHC-I in infected cells [71]. In contrast, antigen-specific CD8^+^ T-cell cytolytic activity on infected target cells is thought to play a crucial role in disease recovery, given that depletion of CD8^+^ T-cells resulted in an increased viral burden in the CNS [40]. Supportively, our data revealed that IDO ablation provided marked enhancement of CD8^+^ T-cells producing IFN-γ and TNF-α upon JEV Ag stimulation. In addition, IDO ablation induced an increase in CD4^+^ Th1 cells producing IFN-γ at the early stage, but IL-17^+^CD4^+^ Th17 and CD4^+^Foxp3^+^ Tregs were not altered by the ablation of IDO. Collectively, these findings suggest that IFN-γ produced from CD4^+^ Th1 and CD8^+^ T-cells play an important role in regulating JE progression in IDO-ablated mice.

The role of IDO in antiviral activity has been postulated in different in vitro experiments. For example, IDO activity in astrocytes is induced by the TLR3 ligand polyinosinic-polycytidylic acid, which is involved in antiviral function via the exhaustion of the essential amino acid l-TRP in the environment [72]. This finding is inconsistent with our data, but it is notable that this finding was derived from an in vitro model. The intriguing result in the present study was that IDO-ablated CD11c^+^ DCs, including conventional and plasmacytoid DCs, showed potent and rapid IFN-I innate immune responses to JEV infection. Also, KO-KO and KO-WT BM chimeric models showed systemic IFN-β production at levels higher than in WT-WT and WT-KO BM chimeric models. This supports the notion that myeloid cells derived from HSCs, including CD11c^+^ DCs, may dominantly contribute to the rapid IFN-I innate immune response. This result is strengthened by the finding that IDO KO and 1-MT-treated mice showed induction of a markedly increased IFN-I innate response in the spleen following retrovirus infection [21]. Conventional and plasmacytoid DCs exhibited potent and rapid IFN-I innate responses with different dynamic patterns in the expression of PRRs, transcription factors, and ISG genes following JEV infection. These different IFN-I innate responses in both types of CD11c^+^ DCs are thought to be derived from the intrinsic properties of the specific cell type. Although we did not investigate the detailed mechanism by which IDO-ablated CD11c^+^ DCs displayed a potent IFN-I response to JEV infection, our data suggest the involvement of PRRs (RIG-I, MDA5) and transcription factor IRF7 because the expression levels of RIG-I, MDA5, and IRF-7 were rapidly enhanced in IDO-ablated CD11c^+^ DCs. Also, considering that only a small fraction (10–20 %) of myeloid-derived cells are infected by JEV [73], uninfected myeloid-derived cells are thought to contribute substantially to the induction of antiviral ISGs through early stimulation of the STAT1 transcription factor which induces ISG49, ISG54, and ISG56 [24]. Also, it is worth noting that primary cortical neuron cells derived from IDO-ablated mice showed potent IFN-I innate responses resulting in delayed replication of JEV. Although the induction patterns of PRRs and their transcription factors in neuron cells differed from those of CD11c^+^ DCs following JEV infection, the induction of ISGs in IDO-ablated neurons definitely followed potent IFN-I innate immune responses, such as IFN-αβ production. However, because cortical neurons are relatively insensitive to the antiviral effects of IFN-I [74], the regulation of viral replication in primary cortical neurons by IFN-I innate responses may ultimately show some limitations. Indeed, our data indicating that primary cortical neuron cells derived from IDO KO mice showed transient inhibition of viral replication support this speculation. Therefore, further studies are needed to define the mechanistic and functional intermediates that link IDO to the regulation of IFN-I innate immune responses in different cell types.

JE pathogenesis in the murine model may be affected by the route of administration, dosage, and strain of the virus, and the virus propagation conditions used [23]. IDO was expressed with different dynamic patterns in conventional and plasmacytoid DCs, macrophages, and neuron cells upon JEV infection. Thus, it is thought that JE pathogenesis may also be affected by how the innate responses of specific cell types are exhibited after JEV infection. Rapid IFN-I/II innate immune responses are considered more crucial in the control of JE progression than adaptive T-cell responses, which take time to develop. Considering that IDO ablation provided early and enhanced IFN-I/II innate responses and subsequent adaptive T-cell response in the host, the inhibition of IDO may be a valuable tool for regulating JE progression.

## Conclusions

The inhibition of IDO ameliorates JE progression via rapid enhancements in IFN-I/II innate immune responses and CNS infiltration by Ly-6C^hi^ monocytes. Also, IDO ablation may accelerate viral clearance from the host at a later stage via the enhancement of JEV-specific IFN-γ-producing T-cell responses. Therefore, these results suggest that inhibition of IDO activity by specific inhibitors [75, 76] may be a promising tool for therapeutic and prophylactic strategies against JE.

## Abbreviations

BBB: blood-brain barrier
BM: bone marrow
BMDC: bone marrow-derived dendritic cell
BMDM: bone marrow-derived macrophage
dpi: days post-infection
HSC: hematopoietic stem cell
IDO: indoleamine 2,3-dioxygenase
IFN-I/II: type I/II interferon
JE: Japanese encephalitis
KO: knockout
pDC: plasmacytoid dendritic cell
PRRs: Pattern recognition receptors
WNV: West Nile virus
